# The Anterior Versus Posterior Approach for Interbody Fusion in Patients Who Are Classified as Obese: A Retrospective Cohort Study of 9,021 Patients From a National Database

**DOI:** 10.7759/cureus.44861

**Published:** 2023-09-07

**Authors:** Josette C Graves, Peter G Zaki, Joshua Hancock, Katherine C Locke, Trevor Luck

**Affiliations:** 1 Neurosurgery, Drexel University College of Medicine, Wyomissing, USA; 2 Medicine, Drexel University College of Medicine, Wyomissing, USA; 3 Neurological Surgery, University at Buffalo, Buffalo, USA; 4 Orthopedic Surgery, St. Luke's University Health Network, Philadelphia, USA

**Keywords:** posterior fusion, acs-nsqip, obesity, plif, alif

## Abstract

Introduction

Lumbar spine interbody fusions have been performed to relieve back pain and improve stability due to various underlying pathologies. Anterior interbody fusion and posterior interbody fusion approaches are two main approaches that are classically compared. In an attempt to compare these two approaches to the spine, large retrospective national database reviews have been performed to compare and predict 30-day postoperative outcomes; however, they have conflicting findings. Obesity, defined as having a body mass index (BMI) over 30 kg/m^2^, may also contribute to the extent of spine pathology and is associated with increased rates of postoperative complications. Complication rates in patients who are obese have yet to be thoroughly investigated using a large national database. Our present investigation aims to make this comparison using the American College of Surgeons National Surgical Quality Improvement Program (ACS-NSQIP) database. The goal of the present study is to utilize a nationwide prospective database to determine short-term differences in postoperative outcomes between posterior and anterior lumbar fusion in patients with obesity and relate these findings to previous studies in the general population.

Methods

A retrospective cohort analysis was conducted on 9,021 patient data from the ACS-NSQIP database from 2015 to 2019 who underwent an elective, single-level fusion via anterior or posterior surgical approach. This database captures over 150 clinical variables on individual patient cases, including demographic data, preoperative risk factors and laboratory values, intraoperative data, and significant events up to postoperative day 30. All outcome measures were included in this analysis with special attention to rates of deep venous thrombosis (DVT) and pulmonary embolism (PE), prolonged length of stay (LOS), reoperation, and operation time.

Results

Multivariable analysis controlling for age, BMI, sex, race, functional status, American Society of Anesthesiologists (ASA) class, and selected comorbidities with P < 0.05 demonstrated that the anterior approach was an independent predictor for all significant outcomes except prolonged length of stay. Compared to the posterior approach, the anterior approach had a shorter total operation time (B = -13.257, 95% confidence interval (CI) [-17.522, -8.992], P* *< 0.001), higher odds of deep vein thrombosis (odds ratio (OR) = 2.210, 95% CI [1.211, 4.033], P= 0.010), and higher odds of pulmonary embolism (OR = 2.679, 95% CI [1.311, 5.477], P = 0.007) and was protective against unplanned reoperation (OR = 0.702, 95% CI [0.548, 0.898], P* *= 0.005).

Conclusions

The obese population makes up a large and growing demographic of those undergoing spine surgery, and as such, it is pertinent to investigate the differences, advantages, and disadvantages of lumbar fusion approaches in this group. While anterior approaches may be protective of longer operation time and unplanned reoperation, this benefit may not be clinically significant when considering an increased risk of DVT and PE. Given the short-term nature of this dataset and the limitations inherent in large de-identified retrospective database studies, these findings are interpreted with caution. Longer-term follow-up studies accounting for confounding variables with spine-centered outcomes will be necessary to further elucidate these nuances.

## Introduction

Since their introduction, lumbar spine interbody fusions have been performed to relieve back pain and improve stability due to various underlying pathologies [1,2]. There are different approaches based on the surgeon’s preference after considering the patient’s other comorbidities and preferences [2]. Anterior interbody fusion and posterior interbody fusion approaches are two main approaches that are classically compared. Anterior fusions include a variety of incisions and approaches (anterior lumbar interbody fusion (ALIF), oblique lateral interbody fusion (OLIF), direct lateral interbody fusion (DLIF), and extreme lateral interbody fusion (XLIF)) that provide access to the anterior side of the vertebral column [3]. Posterior interbody fusions refer to both transforaminal lumbar interbody fusion (TLIF) and posterior lumbar interbody fusion (PLIF) procedures. With a posterior approach, an incision is made through the lumbosacral fascia to access the vertebral bodies with careful retraction of the spinal nerves [3]. In an attempt to compare these two approaches to the spine, large retrospective national database reviews have been performed to compare and predict 30-day postoperative outcomes; however, they have conflicting findings [1-5]. A sample of 26,336 patients 18 years and older in the general population undergoing either an anterior or posterior approach between the years 2005 and 2015 was studied using the American College of Surgeons National Surgical Quality Improvement Program (ACS-NSQIP) database [1]. Multivariable regression methods examined 30-day readmission, reoperation, and morbidity rates. Posterior approaches were associated with 15.5% increased odds of morbidity, while readmission and reoperation odds were found to be comparable [1]. Older age and higher American Society of Anesthesiologists (ASA) class also predicted worse 30-day postoperative outcomes irrespective of surgical approach [1].

Anterior and posterior approaches have their own benefits and disadvantages for the patients and surgeons [1-5]. While a preferred surgical approach was not universally stated, retrospective studies agree that the decision to perform anterior versus posterior spine operations should be on a case-by-case basis [1,2,5]. Comorbidities linked to spinal pathology, such as obesity, need to be considered in greater detail to make an informed decision on surgical approach [6].

Obesity may contribute to the extent of spine pathology. Obese patients, defined as having a body mass index (BMI) over 30 kg/m^2^, represent 35% of the population of the United States [7]. Furthermore, those classified as either overweight or obese make up about 80% of the population undergoing spine surgery [7]. Obesity is associated with increased morbidity and mortality following various spine surgeries, and there is a significant increase in the surgical complication rate for patients undergoing lumbar spine surgery with a BMI over 35 kg/m^2^ (obesity class 2), with rates further increasing over 40 kg/m^2^ (obesity class 3) [6,7]. Complication rates in patients who are obese have yet to be thoroughly investigated using a large national database. Our present investigation aims to make this comparison using the ACS-NSQIP database.

## Materials and methods

Study design and population

This study is a retrospective cohort analysis of patient data from the ACS-NSQIP database from 2015 to 2019. This database captures over 150 clinical variables on individual patient cases, including demographic data, preoperative risk factors and laboratory values, intraoperative data, and significant events up to postoperative day 30. Given the de-identified nature of this dataset, this study was exempt from institutional review board (IRB) review.

**Figure 1:**
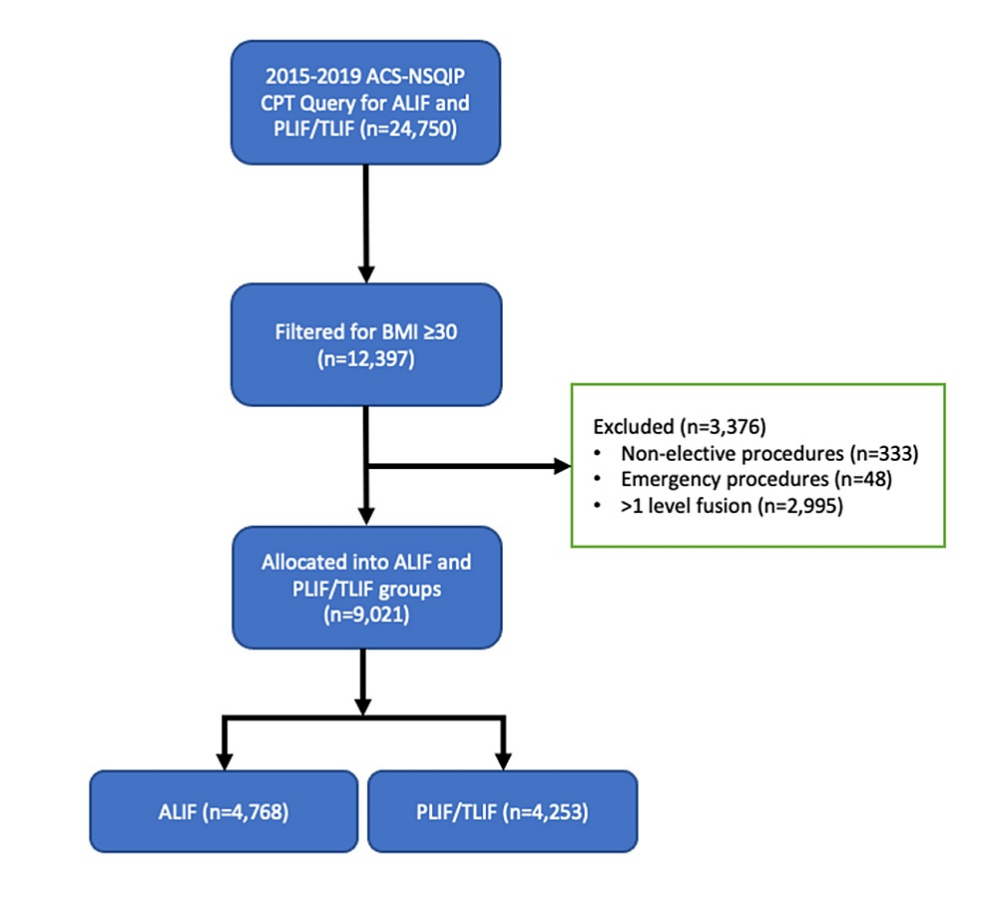
Patient selection ACS-NSQIP: American College of Surgeons National Surgical Quality Improvement Program, n: number of patients, CPT: Current Procedural Terminology, ALIF: anterior lumbar interbody fusion, PLIF/TLIF: posterior lumbar interbody fusion/transforaminal lumbar interbody fusion, BMI: body mass index

The ACS-NSQIP 2015-2019 database was queried for Current Procedural Terminology (CPT) codes 22558 (arthrodesis, anterior interbody technique, including minimal discectomy to prepare interspace, other than for decompression) and 22630 (arthrodesis, posterior interbody technique, including laminectomy and/or discectomy to prepare interspace, other than for decompression, single interspace). Further filters were applied to isolate obese patients defined by a BMI greater than 30 kg/m^2^. Additionally, non-elective procedures and emergency procedures were excluded, while single-level fusions were selected to make outcomes comparisons between the two general approaches. After all these considerations, the total sample size was 9,021 patients (Figure 1). 

Since the goal of this study was to build on the current literature concerning anterior versus posterior lumbar fusions as applied to obese patients, all outcome measures captured by the ACS-NSQIP dataset were considered in this analysis. Given previously published results in the general population, special attention was paid to rates of deep venous thrombosis (DVT) and pulmonary embolism (PE), prolonged length of stay (LOS), reoperation, and operation time [1,5].

Statistical analysis

All calculations and statistics were performed using Statistical Package for the Social Sciences (SPSS) version 26.0 (IBM Corporation, Armonk, NY, USA). Categorical variables are expressed as frequencies and proportions, while continuous variables are expressed as means and standard deviations (SD). Univariable analyses were done on patient characteristics, comorbidities, and 30-day outcomes data using the chi-squared test for categorical variables and the independent samples t-test for continuous variables. Characteristics and comorbidities that were significantly different at P < 0.05, along with clinically important variables such as sex and smoking status, were gathered for multivariable analysis of outcomes data to establish independent predictability of anterior approaches with posterior approaches as the reference.

In multivariable analysis, both binary logistic regression and linear regression were used for categorical variables and continuous variables, respectively. Binary logistic regressions are reported with odds ratio (OR) and 95% confidence intervals (CI), while linear regression was reported with mean difference (B) and 95% CI. Tests were considered significant at P < 0.05.

## Results

Demographic and clinical characteristics

Patient characteristics and comorbidities are presented in Table 1. A total of 9,021 patients were identified. Subjects were 55.9 ± 13.0 years old in the anterior approach group compared to 58.1 ± 12.7 years old in the posterior approach group (P* *< 0.001). There was no significant difference in sex between the two groups (52.7% female in the anterior approach group compared to 54.4% female in the posterior approach group (P* *= 0.106)). There was a significant difference in ethnicity in the two groups (the most common was White at 81% in the anterior group compared to 74.7% in the posterior group, followed by African American at 11.5% in the anterior group compared to 10.9% in the posterior group (P* *< 0.001)). Patients undergoing the posterior approach were more likely to be elderly (≥65 years old) (33.9% versus 28.9%, P* *< 0.001). Both groups had a similar distribution of BMI, sex, functional status, and smoking status (Table 1). ASA classification distribution was significantly different between the two groups (P= 0.041). The posterior approach patients were more likely to have comorbidities including diabetes (24.4% versus 20.9%, P* *< 0.001) and hypertension (61.8% versus 56.5%, P* *< 0.001).

**Table 1:** Patient characteristics and comorbidities *Significant P value N: number of patients, SD: standard deviation, BMI: body mass index, ASA: American Society of Anesthesiologists, non-IDDM: non-insulin-dependent diabetes mellitus, IDDM: insulin-dependent diabetes mellitus

Parameter	Anterior approach (N = 4,768)	Posterior approach (N = 4,253)	P value
Mean age (years±SD)	55.88±12.98	58.09±12.72	<0.001*
Elderly (≥65 years old)	1,376 (28.9%)	1,442 (33.9%)	<0.001*
Sex			0.106
Male	2,256 (47.3%)	1,940 (45.6%)	
Female	2,512 (52.7%)	2,313 (54.4%)	
Race			<0.001*
White	3,864 (81%)	3,179 (74.7%)	
African American	549 (11.5%)	464 (10.9%)	
Asian	35 (0.7%)	40 (0.9%)	
Native American	21 (0.4%)	22 (0.5%)	
Native Hawaiian/Pacific Islander	9 (0.2%)	11 (0.3%)	
Unknown	290 (6.1%)	537 (12.6%)	
Functional status			0.963
Independent	4,681 (98.2%)	4,174 (98.1%)	
Partially dependent	68 (1.4%)	51 (1.4%)	
Totally dependent	3 (0.1%)	4 (0.1%)	
Unknown	16 (0.3%)	14 (0.3%)	
Mean BMI, (kg/m^2^±SD)	35.41±4.73	35.59±4.86	0.063
ASA classification			0.041*
1	81 (1.7%)	69 (1.6%)	
2	2,116 (44.4%)	1,794 (42.2%)	
3	2,469 (51.8%)	2,271 (53.4%)	
4	99 (2.1%)	110 (2.6%)	
5	0 (0%)	0 (0%)	
Not assigned	3 (0.1%)	9 (0.2%)	
Smoking (past year)	872 (18.3%)	739 (17.4%)	0.259
Diabetes			
Non-IDDM	679 (14.2%)	682 (16%)	
IDDM	319 (6.7%)	354 (8.3%)	
Total diabetes	998 (20.9%)	1,036 (24.4%)	<0.001*
Open wound or wound infection	5 (0.1%)	8 (0.2%)	0.298
Hypertension	2,695 (56.5%)	2,630 (61.8%)	<0.001*

Univariable analysis

Univariable analysis of outcomes data is presented in Table 2. Anterior approaches had a shorter operation time on average (191.4 ± 111.1 minutes versus 206.7 ± 92.0 minutes, P* *< 0.001), lower rates of unplanned reoperation (2.4% versus 3.4%, P* *= 0.005), and length of hospital stay ≥ 3 days (51.4% versus 57%, P* *< 0.001). The anterior approach had higher rates of deep venous thrombosis (0.8% versus 0.4%, P* *= 0.008), pulmonary embolism (0.7% versus 0.2%, P* *= 0.003), and unplanned reintubation (0.4% versus 0.1%, P* *= 0.022) compared to the posterior approach.

**Table 2:** Univariable analysis of perioperative outcomes and complications *Significant P value **Prolonged intubation defined as mechanical ventilation greater than 48 hours N: number of patients, SD: standard deviation, LOS: length of stay, SSI: surgical site infection

Parameter	Anterior approach (N = 4,768)	Posterior approach (N = 4,253)	P value
Operation time (minutes±SD)	191.42±111.12	206.74±91.97	<0.001*
Total LOS (days±SD)	3.13±4.96	3.22±3.62	0.309
Superficial SSI	62 (1.3%)	71 (1.7%)	0.147
Deep SSI	19 (0.4%)	22 (0.5%)	0.402
Organ space SSI	19 (0.4%)	16 (0.4%)	0.865
Wound dehiscence	18 (0.4%)	16 (0.4%)	0.992
Acute postoperative renal failure	9 (0.2%)	3 (0.1%)	0.124
Bleeding requiring transfusion	179 (3.8%)	173 (4.1%)	0.443
Cardiac arrest	6 (0.1%)	4 (0.1%)	0.651
Deep vein thrombosis	37 (0.8%)	15 (0.4%)	0.008*
Pulmonary embolism	31 (0.7%)	10 (0.2%)	0.003*
Myocardial infarction	12 (0.3%)	10 (0.2%)	0.874
Pneumonia	22 (0.5%)	25 (0.6%)	0.405
Sepsis	29 (0.6%)	18 (0.4%)	0.223
Septic shock	5 (0.1%)	4 (0.1%)	0.871
Unplanned reintubation	17 (0.4%)	5 (0.1%)	0.022*
Urinary tract infection	61 (1.3%)	61 (1.4%)	0.525
Prolonged intubation**	1 (0.1%)	0 (0%)	0.345
Non-home discharge	564 (11.8%)	559 (13.1%)	0.059
Unplanned reoperation	116 (2.4%)	146 (3.4%)	0.005*
LOS ≥ 3 days	2,451 (51.4%)	2,426 (57%)	<0.001*
Still in hospital >30 days	8 (0.2%)	2 (0.1%)	0.085
Readmission	229 (4.8%)	205 (4.8%)	0.969

Multivariable analysis

Multivariable analysis is presented in Table 3. Controlling for age, BMI, sex, race, functional status, ASA class, and selected comorbidities with P* *< 0.05, the anterior approach was found to be an independent predictor for all significant outcomes except prolonged length of stay. Compared to the posterior approach, the anterior approach had a shorter total operation time (B = -13.257, 95% CI [-17.522, -8.992], P* *< 0.001), higher odds of deep vein thrombosis (OR = 2.210, 95% CI [1.211, 4.033], P= 0.010), and higher odds of pulmonary embolism (OR = 2.679, 95% CI [1.311, 5.477], P* *= 0.007) and was protective against unplanned reoperation (OR = 0.702, 95% CI [0.548, 0.898], P* *= 0.005).

**Table 3:** Multivariable analysis of outcomes The posterior approach was used as a reference point when compared to the anterior approach. Operation time was analyzed using multivariable linear regression, while pulmonary embolism and total length of stay greater than or equal to three days were analyzed via backwards conditional binary logistic regression analysis. Data was controlled for age, race, diabetes, and hypertension requiring medication. *Significant P value B: mean difference for linear regression, OR: odds ratio for binary logistic regression, ^a^: mean difference for linear regression (B) calculation, ^b^: odds ratio for binary logistic regression (OR) calculation, CI: confidence interval

Outcome	B or OR	95% CI	P
Operation time	-13.257^a^	[-17.522, -8.992]	<0.001*
Deep venous thrombosis	2.21^b^	[1.211, 4.033]	0.010*
Pulmonary embolism	2.679^b^	[1.311, 5.477]	0.007*
Unplanned reoperation	0.702^b^	[0.548, 0.898]	0.005*

## Discussion

Comparison to general population outcomes

Patients with obesity make up a significant part of the population as a whole and even more so of the spinal surgery population [6,8]. These data show that when compared to posterior approaches, anterior approaches are associated with marginally higher rates of DVT and PE, unplanned reoperation, and shorter operative times.

Higher rates of DVT/PE in the anterior approach are consistent with a general population study examining all patients undergoing anterior versus posterior approaches [9]. DVTs and arterial thrombi are thought to be more prominent in anterior lumbar fusions due to the constant handling and compression of major abdominal vessels. Many have blamed the Steinmann pin and fixed abdominal retractor for these complications [10-13].

Moreover, one study in the general population found that patients undergoing the anterior approach were 1.6 times more likely to sustain a DVT postoperatively than those undergoing a posterior approach [2]. The present study suggests that obesity may be 2.2 times more likely for DVT and 2.6 times more likely for PE. While the frequency of these complications remain low, our findings suggest that patients with obesity may be at even higher risk for these complications.

The anterior approach was found to be protective against unplanned reoperation and had a shorter operative time of about 13 minutes (P* *< 0.001). These are important from both a financial and resource allocation perspective. While patients with obesity had 0.7 times the risk of reoperation with the anterior approach, this benefit may not outweigh the higher risk of venous thrombi/emboli. While the anterior approach did have a shorter operative time and was consistent with other studies in the literature [5], a real difference of 13 minutes for a single level fusion may not be clinically relevant. To what extent this time difference might be cost-effective is questionable at best. In an inpatient setting at a large academic center, much of the time and cost variation may be more dependent on factors extrinsic to the actual procedure length (e.g., staffing concerns, disruptions to the operating room schedule, and time for medical optimization prior to surgery).

Benefits and limitations

The ACS-NSQIP is a national database that includes a large sample size of claims data, which is advantageous in retrospective surgical studies [8,9]. Heterogenous samples and populations that may have not qualified for a randomized control trial may be examined in registry-based data [8]. However, several important limitations exist when using large, de-identified databases such as the ACS-NSQIP.

Firstly, given the retrospective nature of data collection using registry data, there is a danger of missing data and recall bias that is seen less in prospective research with randomized controlled trials [8]. Additionally, methodically handling patients lost to follow-up is critical as these patients may have systematically different outcomes than compliant patients [8]. These claims data also have the potential to include incorrect CPT codes due to billing errors. Unlike single-institution studies, retrospective analyses of multicenter de-identified databases do not have the privilege of data verification by chart review. In addition, with a retrospective study, only associations can be identified, and it is not possible to establish causality. In particular, the odds ratios reported by this study are not to be interpreted as relative risk, although with a sample size greater than 100, they might approximate it [14].

Secondly, the ACS-NSQIP database also captures limited information regarding past medical and surgical history. Some common elements of the medical history are elucidated, such as the presence of hypertension and diabetes, but the severity (i.e., systolic blood pressure or last hemoglobin A1C) is not clear. Prescribed medications that would be relevant to bleeding risk and wound healing such as antiplatelets and anticoagulants are omitted. Several elements are also no longer captured, such as chemotherapy treatment and stroke history. Relevant surgical history such as bariatric surgery and previous spine surgery is not included. These are but a few data points among many that may be omitted in large registries such as the ACS-NSQIP, and as such, a multitude of hidden confounders may cloud our results and limit the ability to fully appreciate the complete clinical picture. Despite this, many studies still make use of this dataset, given that the large sample size of the dataset should provide enough power to draw meaningful conclusions [8].

Thirdly, another limitation of this dataset is that only 30-day, general postsurgical complications are captured, and as such, the testable hypotheses with this dataset are limited and somewhat nonspecific to spine surgery. While deep venous thrombosis and pulmonary embolism are two complications included in the database, the ACS-NSQIP does not address outcomes that may be more unique to an anterior approach, such as retrograde ejaculation [15]. Spine-specific outcomes such as fusion rates, angle of lordosis, Oswestry Disability Index, and pain score are also omitted. Strangely, postoperative ileus, a common complication, is also not captured by the dataset. While this dataset has strength in generalized outcomes studies due to its quality control and sample size, it is clearly lacking in specificity to certain procedures. While the ACS-NSQIP program does have several procedure-focused datasets such as the appendectomy participant use file, it has none for spine procedures [16].

For the purposes of this study, only one level of fusion was included for the sake of removing levels fused as a confounding variable. However, the choice to focus on only one level sacrifices nuance inherent in multiple levels of fusion or the spinal level being fused. An anterior approach is often only viable for L5-S1 levels of fusion due to the working space between the iliac vessels, and it is atypical for surgeons to expose levels higher than L2, both due to difficulty navigating around the aorta and retroperitoneal viscera [15]. A posterior approach is preferred for higher lumbar fusions in the L1-L2 region, regardless of other factors [3,4]. Any effect of these differences in spinal levels and preference is lost by looking at single levels.

To curb some of the limitations presented, future studies should create prospective, multicenter databases with longer-term follow-up and collect outcomes data that are relevant to the procedures of interest. Several of these databases already exist, such as the American Spine Registry. However, access is limited depending on institutional membership [17]. These registries will not only fix some of the glaring limitations in current studies, such as the aggregation of posterior approaches due to the reliance on CPT coding, but they will also ameliorate this underlying lack of specificity by way of incorporating data points such as imaging studies, intraoperative variables such as the inclusion of robotics, specific procedure coding including for minimally invasive approaches, and long-term follow-up information such as persistence of pain.

## Conclusions

There are many factors to consider when deciding between anterior and posterior approaches that vary among patients and operating physicians. The obese population makes up a large and growing demographic of those undergoing spine surgery, and as such, it is pertinent to investigate the differences, advantages, and disadvantages of lumbar fusion approaches in this group. While anterior approaches may be protective of longer operation time and unplanned reoperation, this benefit may not be clinically significant when considering an increased risk of DVT and PE.

Given the short-term nature of this dataset and the limitations inherent in large de-identified retrospective database studies, these findings should be interpreted with caution. While studies done in the general population have also shown a higher rate of DVT with anterior approaches, to what extent this association is clinically significant remains questionable. Longer-term follow-up studies accounting for confounding variables with spine-centered outcomes will be necessary to further elucidate these nuances.

## References

[1] Katz AD, Mancini N, Karukonda T, Greenwood M, Cote M, Moss IL (2019). Approach-based comparative and predictor analysis of 30-day readmission, reoperation, and morbidity in patients undergoing lumbar interbody fusion using the ACS-NSQIP dataset. Spine (Phila Pa 1976).

[2] Qureshi R, Puvanesarajah V, Jain A, Shimer AL, Shen FH, Hassanzadeh H (2017). A comparison of anterior and posterior lumbar interbody fusions: complications, readmissions, discharge dispositions, and costs. Spine (Phila Pa 1976).

[3] Coric D, Rossi VJ, Kim PK (2017). Youmans and Winn neurological surgery, seventh edition.

[4] Mobbs RJ, Phan K, Malham G, Seex K, Rao PJ (2015). Lumbar interbody fusion: techniques, indications and comparison of interbody fusion options including PLIF, TLIF, MI-TLIF, OLIF/ATP, LLIF and ALIF. J Spine Surg.

[5] Upadhyayula PS, Curtis EI, Yue JK, Sidhu N, Ciacci JD (2018). Anterior versus transforaminal lumbar interbody fusion: perioperative risk factors and 30-day outcomes. Int J Spine Surg.

[6] Sheng B, Feng C, Zhang D, Spitler H, Shi L (2017). Associations between obesity and spinal diseases: a Medical Expenditure Panel study analysis. Int J Environ Res Public Health.

[7] Bono OJ, Poorman GW, Foster N (2018). Body mass index predicts risk of complications in lumbar spine surgery based on surgical invasiveness. Spine J.

[8] Claus CF, Lytle E, Carr DA, Tong D (2021). Big data registries in spine surgery research: the lurking dangers. BMJ Evid Based Med.

[9] Schoenfeld AJ (2015). Spine surgical research: searching for absolute truth in the era of "big data". Spine J.

[10] Vint H, Mawdsley MJ, Coe C, Jensen CD, Kasis AG (2021). The incidence of venous thromboembolism in patients undergoing anterior lumbar interbody fusion: a proposed thromboprophylactic regime. Int J Spine Surg.

[11] Watkins R (1992). Anterior lumbar interbody fusion surgical complications. Clin Orthop Relat Res.

[12] Baker JK, Reardon PR, Reardon MJ, Heggeness MH (1993). Vascular injury in anterior lumbar surgery. Spine (Phila Pa 1976).

[13] Ikard RW (2006). Methods and complications of anterior exposure of the thoracic and lumbar spine. Arch Surg.

[14] Ranganathan P, Aggarwal R, Pramesh CS (2015). Common pitfalls in statistical analysis: odds versus risk. Perspect Clin Res.

[15] Xu DS, Walker CT, Godzik J, Turner JD, Smith W, Uribe JS (2018). Minimally invasive anterior, lateral, and oblique lumbar interbody fusion: a literature review. Ann Transl Med.

[16] (2023). ACS NSQIP participant use data file. http://www.facs.org/quality-programs/acs-nsqip/participant-use.

[17] (2023). American Spine Registry. https://www.americanspineregistry.org/.

